# Is Protein BLAST a thing of the past?

**DOI:** 10.1038/s41467-023-44082-5

**Published:** 2023-12-11

**Authors:** Ali Al-Fatlawi, Martin Menzel, Michael Schroeder

**Affiliations:** 1 Biotechnology Center (BIOTEC), Center for Molecular and Cellular Bioengineering, Technische Universität Dresden, Dresden, Germany; 2 Center for Scalable Data Analytics and Artificial Intelligence (ScaDS.AI), Dresden, Germany

**Keywords:** Computational biology and bioinformatics, Bioinformatics, Evolution

## Abstract

Will protein structure search tools like AlphaFold replace protein sequence search with BLAST? We discuss the promises, using structure search for remote homology detection, and why protein BLAST, as the leading sequence search tool, should strive to incorporate structural information

## Main

BLAST^1^ is widely used in molecular biology to search for nucleotide and protein sequences. Three decades after BLAST was introduced, there were major breakthroughs in structure prediction, and tools such as RoseTTAFold^2^ and AlphaFold^3^ emerged. Consequently, every protein sequence in the major sequence databases now comes with a model of how it folds in 3D. While this does not affect (non-coding) nucleotide sequences, it begs the question of whether a search over 3D protein structures will replace a search over protein sequences. Is Protein BLAST a thing of the past?

While BLAST searches are a powerful tool in function prediction, they are limited. Sequences can degrade significantly and still fold into similar 3D structures that perform the same or similar functions.

### Different sequences, same structures

**Fig. 1:**
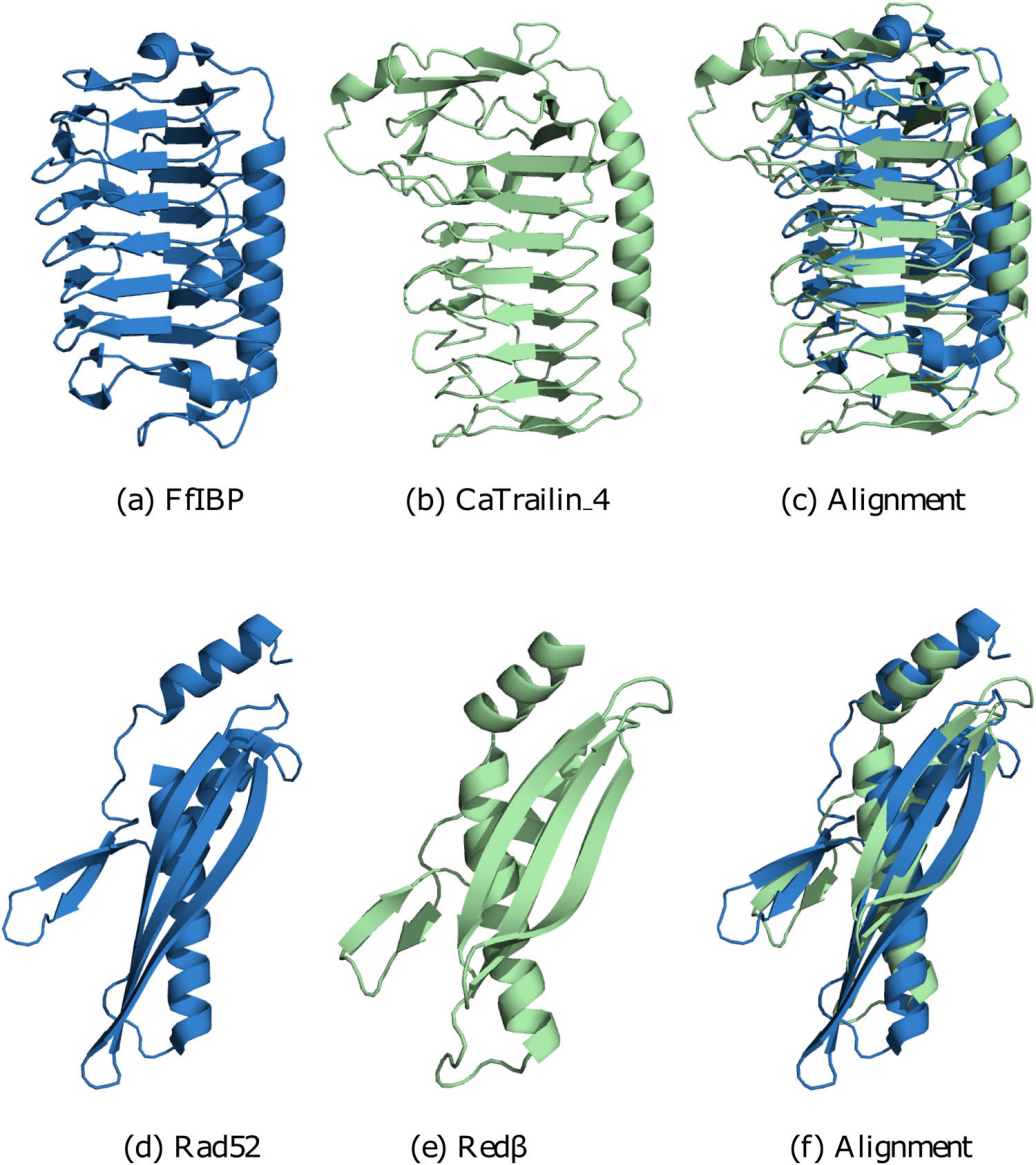
FfIBP (a)/CaTrailin_4 (b) and Rad52 (d)/Red*β* (e)  have a poor E-value around 0.3. Yet, their structures superpose very well (**c**, **f**), suggesting that they may be distant homologues.

An impressive example of such a protein pair can be found in adhesion molecules of algae and bacteria^4^, specifically in the diatom adhesion protein CaTrailin_4 and the bacterial ice-binding protein FfIBP. The pair has no sequence similarity detectable by BLAST (E-value 0.30, where E-values > 0.001 are not considered significant). In fact, even more refined sequence-based tools such as HHblits^5^ cannot establish a relation, either. Yet, the predicted structure of CaTrailin_4 and the known structure of FfIBP resemble each other closely as both adopt a beta helical fold consisting of two units held by an alpha helix - a topology characteristic for ice-binding proteins^4^ (see Fig. 1a–c).

Such structural similarities can be measured by the so-called template modelling score (TM-score), which combines RMSD (root mean square deviation) and alignment length in an interpretable score. A TM-score greater than 0.5 implies that two structures are likely to adopt the same fold^6,7^ and are evolutionarily related. For an independent evaluation of this cut-off, see Supplementary Note 2. A TM-score of 0.6—above the 0.5 cut-off—is achieved for CaTrailin_4 and FfIBP. Thus, structure comparison can uncover this striking similarity, which remains elusive for BLAST and other sequence-based tools such as HHblits.

Another example concerns DNA recombination, a fundamental process in replication in which single-strand annealing proteins (SSAP) play a central role. For more than two decades, it has been suspected and controversially discussed whether RecT/Red*β*, ERF, and RAD52 form three different or just one superfamily. The former view is supported by sequence analysis which shows no demonstrable similarity between RecT/Red*β*, ERF, and RAD52. In fact, Rad52 and Red*β* have no similarity detectable by BLAST (E-value 0.38). Taking structure into account changes the picture. Al-Fatlawi et al. juxtapose representative structures of RecT/Red*β*, ERF, and RAD52 side by side and show that despite the lack of sequence similarity, the structures contain one core structural element^8^. It is central in oligomerization as it generates a ring and helix structure, respectively. Consequently, it is very well conserved across RecT/Red*β*, ERF, and RAD52, and it is detectable by structural similarity (TM-score of 0.5) despite the lack of any sequence similarity (see Fig. 1d–f).

### Structure prediction to the rescue

These examples suggest that AlphaFold may be able to step in where BLAST cannot find significant similarity. Hence, the question arises: How can this be achieved systematically? To this end, there are tools such as Foldseek^9^, DALI^10^, and 3D-AF-Surfer^11^, which scan and compare structures using autoencoders, distance matrix alignment, and dedicated fingerprints, respectively. While these tools exist, they still need to be more widespread and straightforward enough to compete with BLAST searches over sequence databases. A synergy is needed that integrates them into a classic BLAST sequence search. A first step in this direction has been recently taken by a study comparing reciprocal best BLAST hits and reciprocal best structural hits^12^ and by nearest neighbour search on machine learning embeddings of sequences^13^.

To explore the potential of such an advanced tool, we wanted to understand how membership in the same superfamily is linked to sequence and structure similarity. Thus, we obtained 11,211 domains in 1954 with superfamilies from the SCOPe database^14^. These form 62,278,380 domain pairs, of which 225,931 (0.36%) are in the same superfamily and can hence be considered homologues.

How many of these homologous pairs can be found directly by sequence and by structure, respectively? At an E-value cut-off of 0.001, BLAST recovers 16,300 (7%) out of the 225,931 pairs. Relaxing the cut-off to 1, the number increases to 25,634 (11%). But even at an E-value of < 10, it does not exceed 15%. These figures greatly improve if one considers more sensitive sequence-based methods such as hidden Markov models. In fact, HHblits is able to retrieve 175,682 pairs (78%) under optimal conditions, which is even better than the 164,468 (73%), which are found through structure comparison (TM-score > 0.5).

However, what about the 62,052,449 pairs which are not in the same superfamily? Among these pairs, there are zero, 9,053, and 72,329 with an E-value of less than 0.001, 1, and 10, respectively. HHblits identify among these 25%, while the false detection of structural alignment was limited to below 2%. Expressed as the area under the curve, HHblits achieves an AUC of 77% and the structure comparison 95% compared with 44% in Blast. A higher AUC score indicates that the classifier is more effective at correctly assigning higher scores to proteins in the correct superfamily compared to proteins in other superfamilies. See Supplementary Note 1–3.

As encouraging as the 95% AUC for structure comparison may be, the availability of high-quality structures may be a limitation. It is estimated that 30% of all eukaryotic proteins contain disordered regions of 50 or more consecutive amino acids^15^, which can be expected to be of poor quality in 3D structure prediction. These regions would be amenable to sequence search with BLAST, while they would not be suitable for a direct structural search. To assess how such a large percentage extends to the whole of the AlphaFold database, we computed the average confidence score for all AlphaFold structures. We found that 80% of all AlphaFold structures have a pLDDT confidence score of 70% or better, meaning that they are modelled well with generally good backbone prediction (see Supplementary Note 4). This means that there is substantial structural data available which is of suitable quality.

### BLAST, a thing of the future

BLAST perfectly addresses many needs of biomedical researchers such as detection of variants and closely related sequences. However, the specific problem of remote homology detection is hard for pure sequence search. Here, structure can go much further than sequence^12^. We have evaluated this relationship of sequence and structural similarity by a demonstration analysis of millions of pairs of domains. Taken together, the analysis suggests that BLAST with a stringent E-value is very precise at finding homologues but is not comprehensive. Hidden Markov models are more sensitive but with limited specificity. Structure comparison balances these two extremes. If BLAST search incorporates structural data, it could extend the number of hits which have similar predicted structures and may be candidate homologues without jeopardizing the quality of results.

It is not obvious how to integrate structural data into sequence search, but one approach that appears feasible would be to not use structure data directly but indirectly through so-called embeddings^13^, which are intermediate sequence representations generated by neural networks and which form the basis for structure prediction with neural networks.

However, homology detection building on embeddings and structural data will only contribute to transforming molecular biology if made available in an easy-to-use manner and if widely adopted by the community. Prominent institutes such as the NCBI, EBI, and Riken should now strive to employ fast structure search as implemented in FoldSeek^9^ or the use of embeddings to extend classic BLAST-based protein sequence searches so that Protein BLAST continues to be a thing of the future.

### Supplementary information

Supplementary Information
