# Supplementary material for: Is Protein BLAST a thing of the past?
Source: Nat Commun. 2023 Dec 11;14:8195. doi: 10.1038/s41467-023-44082-5 (PMC10713564; doi:10.1038/s41467-023-44082-5)
Supplement: Supplementary file 1 — Supplementary Information [file 41467_2023_44082_MOESM1_ESM.pdf]

Supplementary Material

1 Supplementary Note 1.

SCOPe40 version 2.01 [1] was accessed in November 2021. Fifty-one genetic domains (indicated by a dot on the side) were removed, leaving 11,211 domains, for which sequences were extracted using BioPython's PDBParser (ignoring HETATM entries). Structures were visualized with PyMol 2.5.0. The SCOPe domains' protein sequences were compared using BLAST 2.8.1+. The reported sequence identity was multiplied by the coverage of the shorter sequence. TM-scores are calculated with USAlign version 20220511 [2]. UniProt IDs FfIBP H7FWB6, Rad532 P43351, and Redβ P03698 were used. CaTrailin_4 is not yet available but has been assigned the ID Q845903.

HH-suite v3.3.0 was used for the HHblits analysis with the UniRef30_2022_02 database. By setting the E-value flag to 0.001, we instructed HHblits to report only those alignments with E-values less than or equal to 0.001.

215 million structures were downloaded on October 2nd, 2022, from the EBI AlphaFold website [3]. pDLLT scores were computed over all atoms per structure.

Balanced accuracy is computed as the average of true positive and negative rates.

All of the data used and generated in this study are publicly available through the following link. This includes PDB files for the FfIBP and CA-Trailin-4 (Figure 1), as well as SCOPe results data, including the alignment scores used in all figures and tables in this manuscript. https://sharing.biotec.tu-dresden.de/index.php/s/GePsiWoy2Rbum9z

Supplementary Note 2: TM-score cut-off of 0.5

A TM-score greater than 0.5 implies that two structures are likely to adopt the same fold [4, 5] and are evolutionary related. We confirmed this cut-off on our own data (see Figure S1). We varied the TM-score cut-off between 0 and 1 and computed true positive, true negative, false positive, false negative rates and balanced accuracy to predict whether a pair of proteins shares the same SCOPe superfamily (see Figure S1). This evaluation was applied to all 62 million protein pairs.

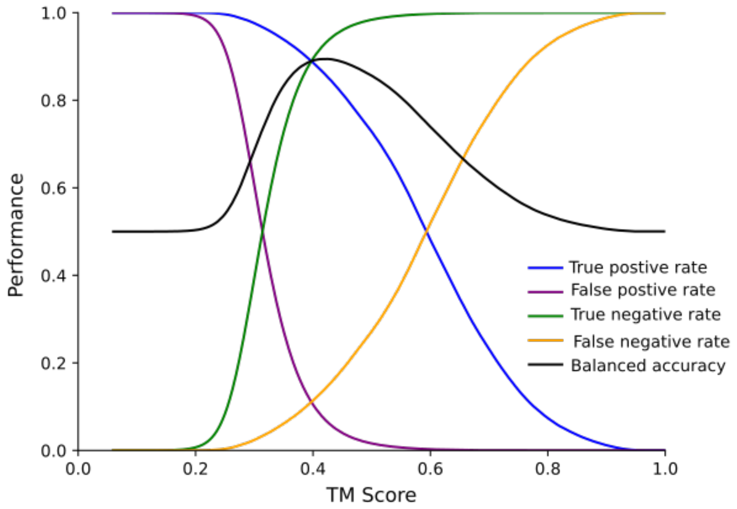

Fig. S1: True positive, true negative, false positive, false negative rates and the balanced accuracy of TM-score to predict shared SCOPe superfamily. A cut-off of 0.4 to 0.5, as reported in the literature, is a good cut-off to separate superfamilies

Supplementary Note 3: HHblits

HHBlits [6] is a widely used alignment tool that uses profile-hidden Markov models (HMMs) to search for homologous protein sequences. We evaluated the performance of HHBlits in the superfamily classification at different thresholds, from 0 to 1 and determined the true positive, true negative, false positive, false negative rates, and balanced accuracy (See Figure S2). The optimal cut-off falls between 15% and 20%. In the last step, we determined the number of protein pairs which are members of the same or different superfamilies according to SCOPe classification. While HHblits demonstrated improved homology inference by detecting 77% of the homologous pairs (175,682 out of 225,931) with an AUC of 77%, it also yielded a false positive above 25% in its best possible cut-off.

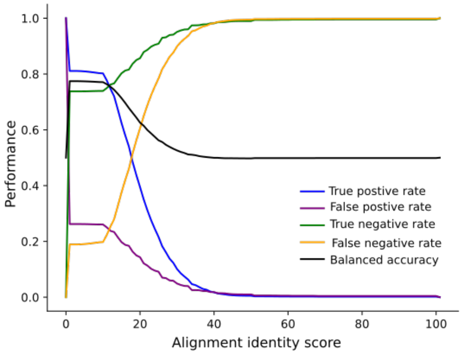

Fig. S2: True positive, true negative, false positive, false negative rates and balanced accuracy and HHblits to predict shared SCOPe superfamily.

Supplementary Note 3: High-quality structures

We analyzed the average confidence of all atoms per predicted AlphaFold structure and found that 80% of them have 70% or higher confidence (pLDDT) (see Figure S3), which implies that they are modeled well with good backbone accuracy. Overall, there are sufficient high-quality predictions that structural data could be included in sequence searches.

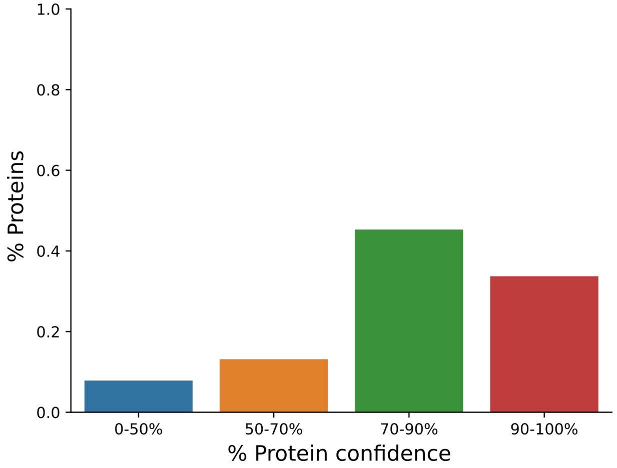

(a)

Fig. S3: Average confidence (pLDDT) per predicted Alphafold structure. 80% of structures have average confidence of 70% or better.

References

[1] Fox, N.K., Brenner, S.E., Chandonia, J.-M.: Scope: Structural classification of proteins—extended, integrating scop and astral data and classification of new structures. Nucleic acids research **42**(D1), 304–309 (2014)

[2] Zhang, C., Shine, M., Pyle, A.M., Zhang, Y.: Us-align: Universal structure alignments of proteins, nucleic acids, and macromolecular complexes. bioRxiv (2022)

[3] Varadi, M., Anyango, S., Deshpande, M., Nair, S., Natassia, C., Yordanova, G., Yuan, D., Stroe, O., Wood, G., Laydon, A., *et al.*: Alphafold protein structure database: massively expanding the structural coverage of protein-sequence space with high-accuracy models. Nucleic acids research **50**(D1), 439–444 (2022)

[4] Xu, J., Zhang, Y.: How significant is a protein structure similarity with TM-score= 0.5? Bioinformatics **26**(7), 889–895 (2010)

[5] Zhang, Y., Skolnick, J.: Tm-align: a protein structure alignment algorithm based on the TM-score. Nucleic acids research **33**(7), 2302–2309 (2005)

[6] Remmert, M., Biegert, A., Hauser, A., Söding, J.: HHblits: lightning-fast iterative protein sequence searching by HMM-HMM alignment. Nature Methods **9**(2), 173–175 (2011). https://doi.org/10.1038/nmeth.1818
